# Supplementary material for: Estimating the Prevalence of De Novo Monogenic Neurodevelopmental Disorders from Large Cohort Studies
Source: Biomedicines. 2022 Nov 9;10(11):2865. doi: 10.3390/biomedicines10112865 (PMC9687899; doi:10.3390/biomedicines10112865)
Supplement: Supplementary file 1 [file biomedicines-10-02865-s001.zip › biomedicines-1882162-supplementary figures.pdf]

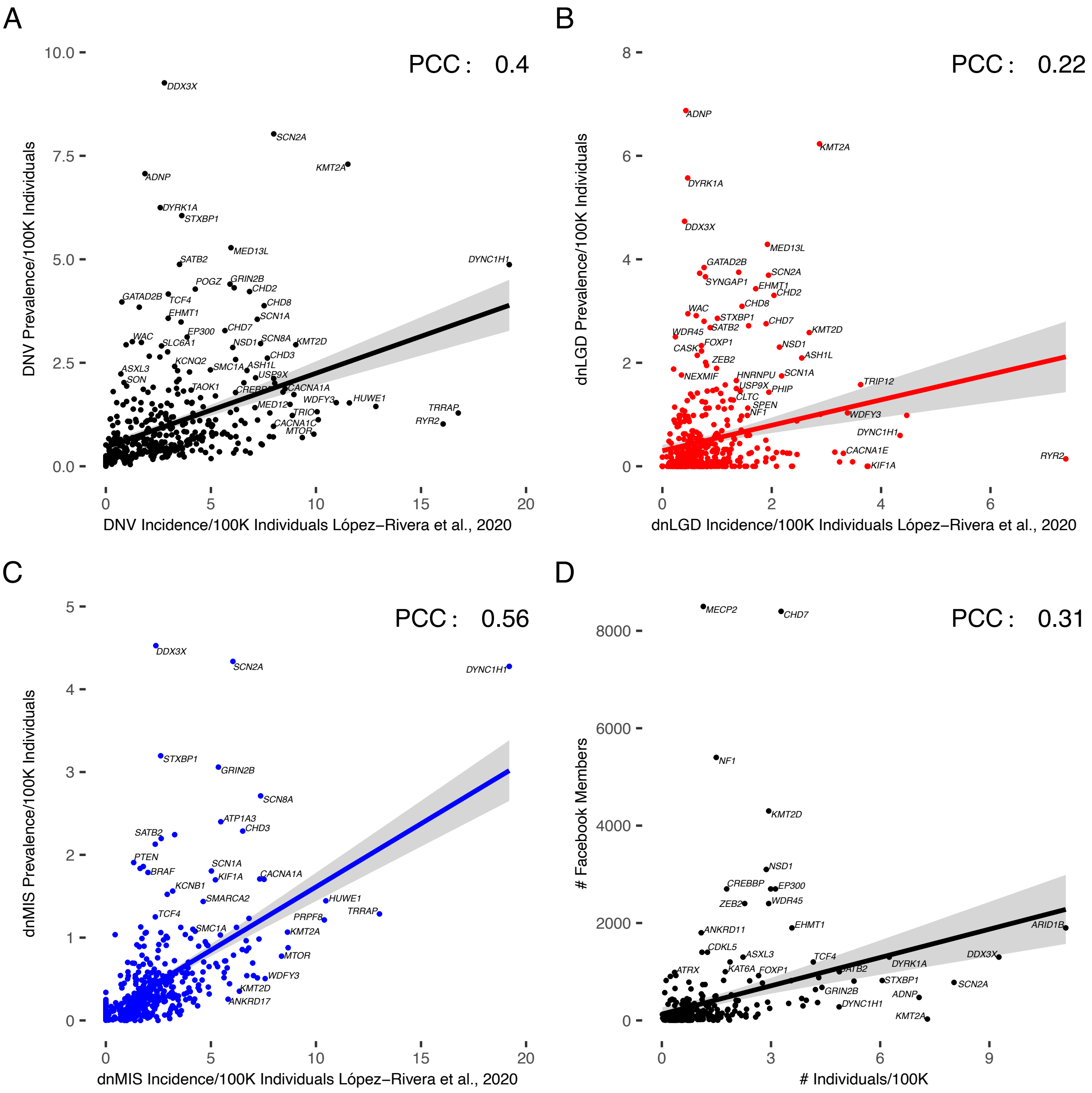

Figure S1. Prevalence of DNVs in candidate NDD genes versus incidence estimates from [14], along with comparison to social media estimates. Confidence intervals are shown in grey. (**A**) NDD DNV cases ($p > 0.0001$ with Bonferroni correction), (**B**) NDD dnLGD cases (not significant), and (**C**) NDD dnMIS cases ($p > 0.0001$ with Bonferroni correction). All mutation types had a positive correlation with previous incidence estimates, shown with Pearson's correlation coefficients (PCC). (**D**) Correlation of prevalence with number of members in gene-focused Facebook groups (not significant). Genes were searched on Facebook if they were in the top 500 of all NDD genes and/or had a named syndrome in OMIM as of July 2022 (n = 293).

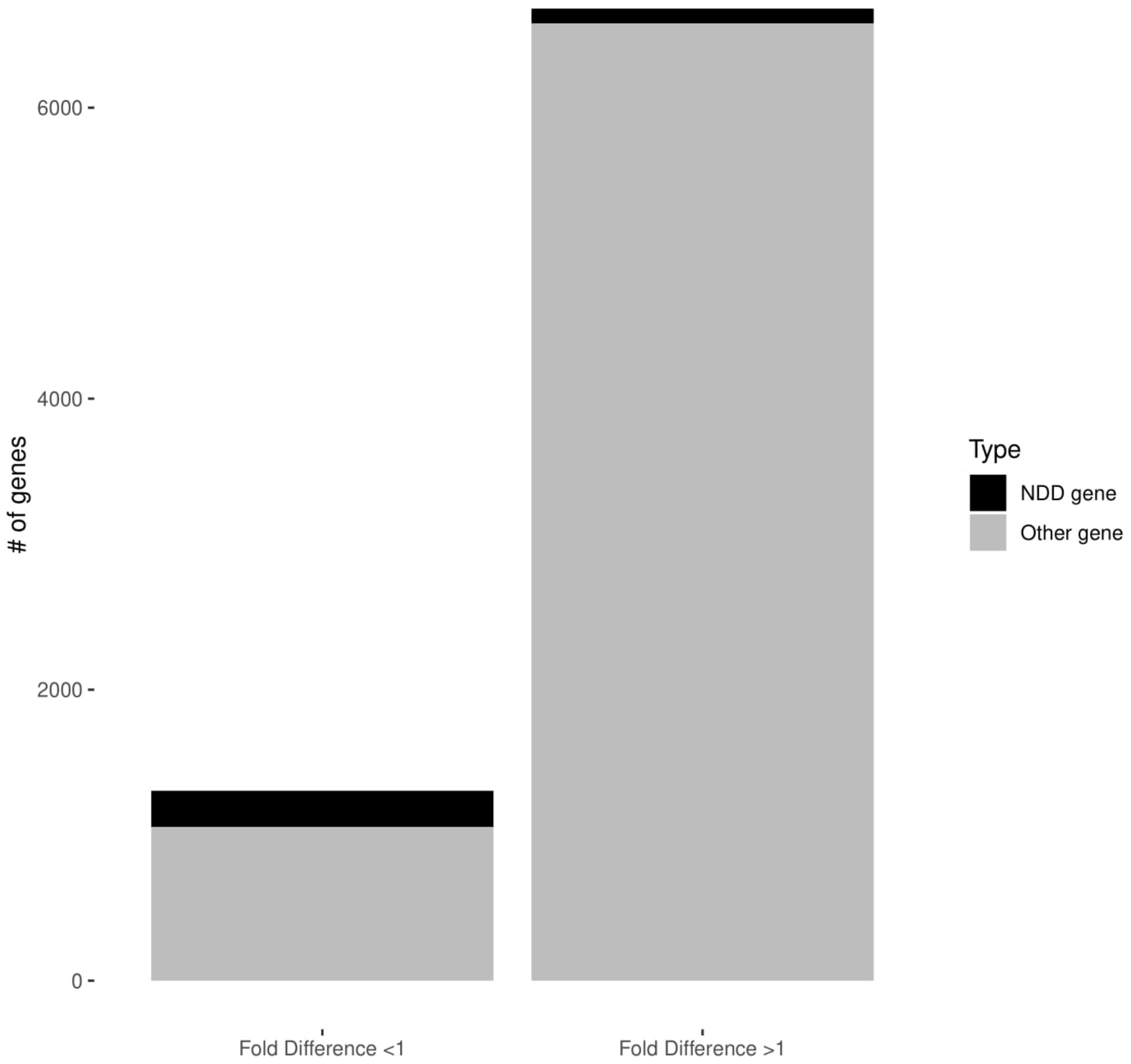

Figure S2. Fold difference between our prevalence estimates and López-Rivera et al.'s incidence estimates. Most genes had a higher prevalence than incidence, as expected. Of the 19% of genes that had lower prevalence than incidence, 1.5% were NDD candidate genes (Chi squared test, $p = 0.0005$).
